# ROOT parameters of sugarcane and soil compaction indicators under deep strip tillage and conventional tillage

**DOI:** 10.1038/s41598-022-21874-1

**Published:** 2022-11-02

**Authors:** Camila Cassante de Lima, Isabella Clerici De Maria, Wellingthon da Silva Guimarães Júnnyor, Getulio Coutinho Figueiredo, Sonia Carmela Falci Dechen, Denizart Bolonhezi

**Affiliations:** 1 Soils and Environmental Resources Center, Agronomic Institute of Campinas (IAC), Av. Barão de Itapura, Campinas, SP 1481 – 13012-970 Brazil; 2 Department of Agronomy, State University of Mato Grosso Do Sul (UEMS), Rod 306 MS, 6.4 Km, Cassilândia, Mato Grosso Do Sul CEP: 79804-970 Brazil; 3 Department of Soils, Bento Gonçalves, Federal University of Rio Grande Do Sul (UFRGS), Porto Alegre, Rio Grande Do Sul 91501-970 Brazil; 4 Sugarcane Center, Agronomic Institute of Campinas (IAC), Rod Antonio Duarte Nogueira, km 321, Ribeirão Preto, SP 14001-970 Brazil

**Keywords:** Plant sciences, Biofuels

## Abstract

Soil tillage and agricultural traffic generate changes in soil physical attributes and affect the growth of the roots. This study evaluates the impact of system soil tillage on compaction and sugarcane root growth. The experiment was carried out on a Rhodic Kandiudox with two soil tillages (Deep Strip Tillage and Conventional Tillage) and two positions (beds or traffic lane and no traffic lane), totaling four treatments (DST-beds + no traffic lane, DST-traffic lane, CT-no traffic lane and CT-traffic lane). Soil penetration resistance (SPR), bulk density, dry mass, and root system lengths and volumes were evaluated. DST-beds presented lower values for SPR (1.45 MPa) compared to the other treatments (2.55 MPa). This lower SPR did not reflect significant increases in root growth in relation to the DST-traffic lane, meaning that the roots were not confined to the beds. The dry root mass for CT- traffic lane was 35% less than for DST- traffic lane, and CT-no traffic lane reduced of the root dry mass in the layers 0.0–0.2 and 0.2–0.4 m by 62% and 47%, respectively, compared to the DST-beds. Therefore, CT, although widely used, does not create adequate conditions for root development in the first sugarcane cycle, even in lanes with no traffic.

## Introduction

Because of socioeconomic, technical, and environmental requirements, the use of mechanized harvesting has become a necessity in Brazil. The increase in mechanized operations for sugarcane production is related to the elimination of the traditional practice of using fire to burn off leaves prior to sugarcane harvesting and has become more common in response to labor shortages^1,2^.

The current process for sugarcane production is based upon the conventional tillage system, which promotes intense mobilization tillage of the surface layer of the soil and soil compaction^3^. Planting and cultivation are performed with evenly spaced single rows of plants, and harvesting is carried out mechanically, with the harvester moving along one row of plants accompanied to one side by a cane collection truck. This leads to heavy vehicular traffic over the field, and the appearance of compacted layers due to the resistance to penetration that negatively affects the root growth of the crop^4^, showed, mainly by the increase of the bulk density and the soil penetration resistance down to the layer of 0.3 m, considered soil compaction indices^5,6^.

In the search for solutions to reduce the soil compaction caused by the heavy traffic of agricultural machines through sugarcane fields, strip tillage systems with auto-guidance farm machinery have emerged as an alternative in traffic control^7^. Within a strip-till system, soil tillage is performed across only a part of the area, forming beds, which are subsequently protected from the passage of machinery, while the areas between the beds are not disturbed. Strip tillage systems seek to minimize the deleterious effects of the passage of farm machinery by separating the fields into zones without traffic, within which the most favorable conditions for root growth can be maintained, and zones where the pressure applied to the soil by the transit of tires is concentrated^8^. In this way, a smaller area of the field is subjected to a traffic pressure compared to conventional tillage, which reduces the impact of soil compaction on the growth of the sugarcane crop^9^.

In this context, the deep strip tillage was adopted in several sugarcane fields in Brazil aiming to reduce soil compaction^10^ and thereby allowing a better development of the culture^11^. However, the lack of understanding about sugarcane root systems is an obstacle to the development of management strategies that promote agricultural production^12,13^.

The distribution of the root system of a sugarcane plant depends on the soil structure, which in turn is related to soil physical attributes^4,14^. However, the effect of soil compaction on the growth and distribution of sugarcane root systems is still poorly understood, and it involves complex mechanisms that are sensitive to many factors^8,15^.

Since each soil tillage system alters the structure of the soil in a manner linked to the degree of applied disturbance, and this may directly affect the root system distribution of the sugarcane crop, further field studies on the distribution of the root systems of sugarcane in deep soil tillage is needed. This research seeks the mitigation of soil compaction, as well as management strategies aimed at optimizing production in a sustainable way.

The hypothesis of this study is that the root systems of sugarcane plants raised in beds prepared by deep strip tillage will be clearly different from those of plants raised under conventional tillage. The aim was to evaluate the impact of two soil tillage systems (deep strip tillage and conventional tillage) on soil compaction indicators and on the development of the root system of sugarcane for one cane planting cycle.

## Materials and methods

### Location and characterization of the experimental area

The experiment was carried out in Piracicaba (state of São Paulo, Brazil) (22° 41' S, 47° 38' W, 550 m.a.s.l.). The climate is subtropical with dry winters, corresponding to Cfa within the Köppen classification^16^. The mean annual temperature and rainfall are 24 °C and 1,273 mm, respectively. The soil of the experimental area has a clay texture (Table 1), and it was classified as a Rhodic Kandiudox according to the USDA taxonomy^17^.

**Table 1:** Soil physical characteristics of the Rhodic Kandiudox.

Layers (m)	Granulometric fractions					Water content		
	Sandy			Silt	Clay	Texture^a^	FC^b^	PWP^c^
	Coarse	Fine	Total			g g^-1^		
	g kg^-1^							
0.0–0.2	164	284	448	127	425	Clay	0.217	0.125
0.2–0.4	104	217	321	102	577	Clay	0.258	0.144
0.4–0.6	88	202	290	89	621	Heavy clay	0.283	0.162
0.6–0.8	97	188	285	95	620	Heavy clay	0.279	0.167
0.8–1.0	95	210	305	97	598	Clay	0.288	0.165

^a^Descriptions were derived from the particle size distributions according to the texture classification scheme of Brazilian Soil Taxonomy System^17^; ^b^water content in field capacity; ^c^water content in permanent wilting point.

The study area had been under sugarcane cultivation since 2005, with conventional tillage, contour-following furrows, and mechanical harvesting. In November 2012, the ratoons of the previous planting were eliminated with an herbicide, and an initial preparation of the area was performed by ploughing and harrowing. From December 2012 to April 2013, the area was cultivated with soybeans.

### Experimental treatments

The experiment was conducted in two blocks, one for each soil tillage treatments: deep strip tillage (DST) and conventional tillage (CT), subdivided into two sub-plots formed by the trafficked and non-trafficked lanes. Each sub-plot had 6,000 m^2^ (120 m length × 50 m width) and was subdivided into three pseudoreplications (the samples from the treatments were obtained from only one sub-plot). This model of the experiment has also been previously used by other researchers^18,19^.

The DST was performed using a tractor-drawn machine that comprised components for simultaneously subsoiling, tilling the surface to break clods, straw windrowing, and the in-row application of limestone and fertilizers. The surface tillage (0.4 m) was accomplished with a rotary hoe with 16 blades on each wheel, and the shanks performed deep subsoiling down to a depth of 0.8 m. The result was rows of soil tilled with a 1.20 m width. According to soil analysis and^20^ recommendations, 2 Mg ha^-1^ at a depth of 0.4 m, and 0.8 Mg ha^-1^ at a depth of 0.8 m of dolomitic limestone were applied in the beds. The tractor for that operation was a New Holland T8.270 with 201 kW.

The CT was performed using a disk harrow (20 disks 0.61 m in diameter) over the entire area and a light harrowing (24 disks 0.61 m in diameter) to break up clods. According to soil analysis and^20^, 2 Mg ha^-1^ of limestone and 0.8 Mg ha^-1^ of gypsum were applied and incorporated with light harrowing (24 disks of 18-inch diameter) using a Massey Ferguson 292.50 with a 77 kW, extended down to a depth that varied between 0.15 and 0.2 m.

The area was furrowed and the sugarcane (variety: ‘IACSP95-5000’) was handmade planted on both plots in August 2013 in a double alternating row pattern defined by two spacings: 0.9 m and 1.5 m. This design established two distinct zones or lanes: one along which there was no transit of machines or implements and another that was used for wheel paths of cultivation and harvesting operations.

In DST system, the lanes along which there was no traffic were the ones prepared by deep tillage, forming beds (BED), while the traffic lanes (TRA) were not tilled. In CT system, since total area were shallow tilled, the lane along which there was no traffic (NTV) was not termed as a bed. Thereby, the four experimental treatments were identified as DST-BED (deep strip tillage bed with no traffic), DST-TRA (deep strip tillage, traffic lane), CT-NTV (conventional tillage, no traffic lane), and CT-TRA (conventional tillage, traffic lane).

### Soil sampling and soil analyses

In August 2014, undisturbed soil samples were collected at the following depths: 0.0–0.2, 0.2–0.4, 0.4–0.6, 0.6–0.8, and 0.8–1.0 m. The samples were withdrawn in metal rings with a height of 0.025 m and a diameter of 0.070 m, in the centers of the lanes without traffic (DST-BED and CT-NTV), and in the centers of the lanes with traffic (DST-TRA and CT-TRA), for bulk density (Bd) evaluation. The Bd was determined by the ratio between soil dry weight and the ring volume^21^.

Soil samples for root biomass were collected at the same times and layers as the soil samples. At each sampling point, a soil core extending down to a depth of 1.0 m was extracted with a probe (Sondaterra®), which had a length of 1.20 m and an internal diameter of 0.055 m. Each core was divided into five segments, corresponding to the following depth ranges: 0.00–0.20, 0.20–0.40, 0.40–0.60, 0.60–0.80, and 0.80–1.00 m. Each segment, with 0.017 m^3^, was placed in a separate labelled plastic bag containing 20 mL of 70% ethyl alcohol diluted to 20% with water, transferred to the laboratory and maintained at 5 ºC for further processing. In the laboratory, the soil adhering to the roots was washed off with running water. The roots were retained in sieves with a mesh size of 1.0 mm, and after washing, they were packaged in plastic pots, and stored at 0 ºC.

**Figure 1:**
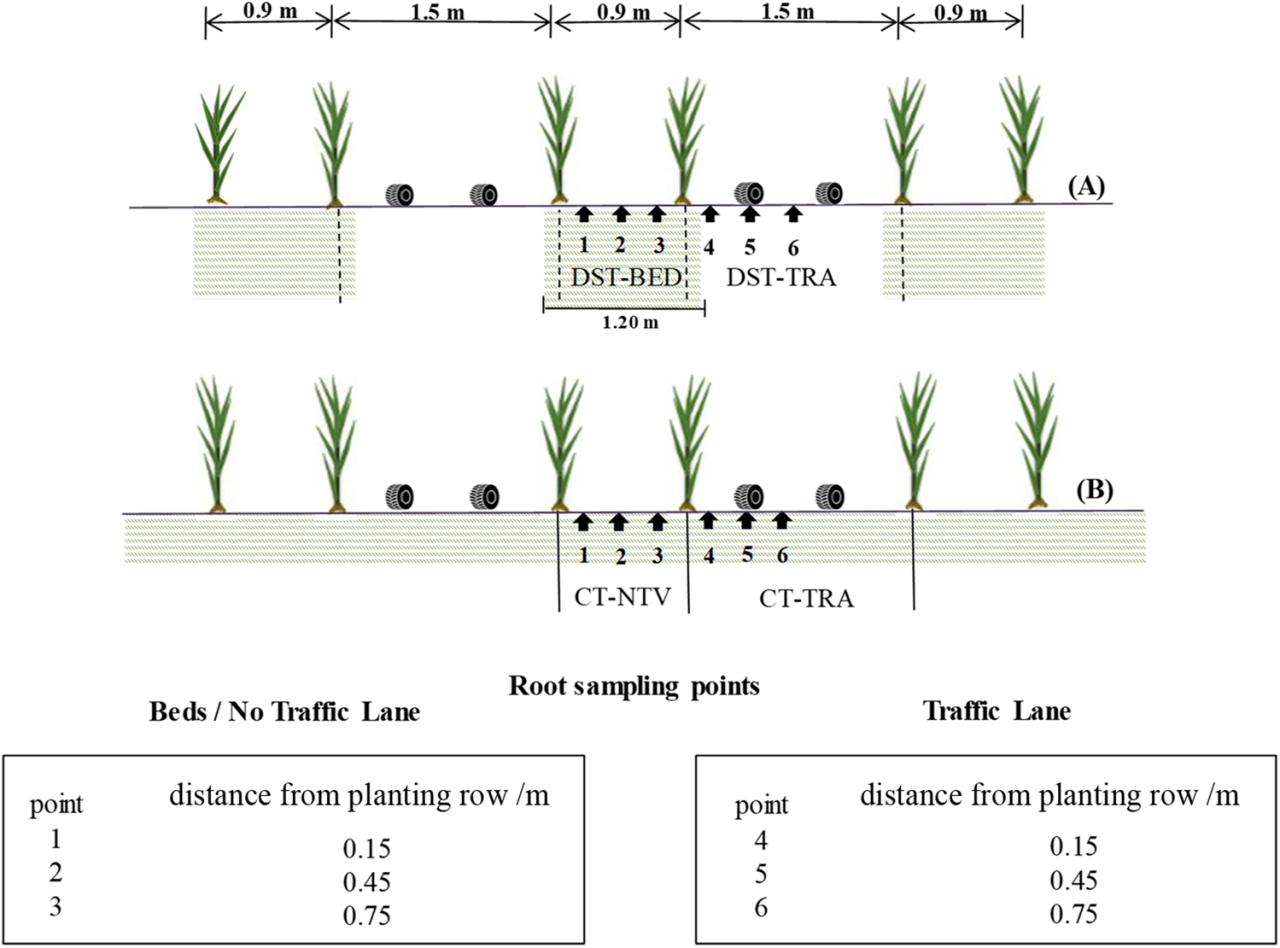
Double row spacing of 0.90 × 1.50 m for sugarcane. (**A**) Deep Strip Tillage (DST) with a tilled and no traffic lane (DST-BED), and a traffic lane (DST-TRA). (**B**) Conventional Tillage (CT) with a no traffic lane (CT-NTV) and a traffic lane (CT-TRA). Hachured areas indicate soil tilled (1,20 m for DTS-BED corresponding to deep strip tillage equipment width). Root sampling points are relative to the planting row. The collected roots comply with relevant institutional, national, and international guidelines and legislation.

The sampling points were in sets of six (Fig. 1): the points were located on lines transverse to the rows of plants at distances of 0.15, 0.45, and 0.75 m from the planting rows, so that three points were within the beds or lanes with no traffic and three were within the lanes with traffic. For each point, three replicate sets of samples were collected.

The root samples were gradually defrosted and arranged over the surface of an acrylic plate in a 5 mm thick film of water for scanning images. The collected images were processed with the software package Safira^22^ to determine total length and volume for the roots in each sample were reported by the software. Following acquisition of the images, the roots were transferred to paper bags and dried in an oven for 72 h at 65 ºC under a flow of air. The dried roots were then weighed to give the dry mass of roots in each sample. We have been granted permission to collect roots from sugarcane (Saccharum officinarum) plants, and reiterate the field research on sugarcane (cultivated plants) and the collection of roots, comply with relevant institutional, national, and international guidelines and legislation.

On November 2014, soil penetration resistance (SPR) was measured with an impact penetrometer (IAA/Planalsucar-Stolf model, manufacture by KAMAQ®), when the soil had moisture at the field capacity (Table 1). The sampling positions for the resistance determinations were the same as previously described for the collection of the root samples, with three replicates. The procedure for the determination of the SPR followed the recommendations of^23^, in which the basis of the calculation is the number of impacts required to drive the point of the instrument down into the soil by a distance of 0.05 m. The SPR tests were effectively conducted every 0.05 m, with the first value assigned to a depth of 0.025 m, down to a depth of 0.6 m; this limit was imposed by the design of the penetrometer. The soil water content was determined over the same pattern of sampling points, with depth intervals of 0.05 m, with a soil moisture sensor probe adapted from the version of^24^.

### Statistical analysis

The data were submitted to tests of normality^25^ and homoscedasticity^26^ using routines from the R statistical software package^27^. Since the data obtained for the water content of the soil, soil penetration resistance, and the root attributes did not display normality and homoscedasticity, a logarithmic transformation defined by y(x) = log([x/units(x)] + 10), was applied to these data to achieve normalization of their variances. The transformed data were inspected for the presence of outliers following a criterion based on the interquartile range (*IQR*). Values outside the range from *Q*_1_—1.5 × *IQR* to *Q*_3_ + 1.5 × *IQR* were considered potential outliers, and the elimination of up to three such values was performed prior to the calculation of the mean and its confidence interval^28^. Data sets were compared through the hypothesis test for difference of means; the difference between two data sets was considered significant when the 85% confidence intervals did not overlap^29^. For the graphical presentation of the results when a log transformation was applied to the raw data, the values for the mean and confidence interval were back-transformed.

## Results

### Soil penetration resistance

**Figure 2:**
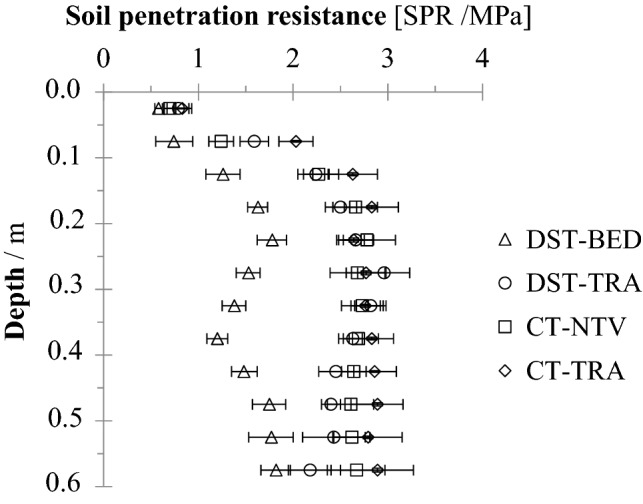
Soil penetration resistance as a function of depth for deep strip tillage bed with no traffic (DST-BED), deep strip tillage traffic lane (DST-TRA), conventional tillage no traffic lane (CT-NTV), and conventional tillage traffic lane (CT-TRA) in a Rhodic Kandiudox. If confidence intervals do not overlap, the differences are significant (p ≤ 0.15).

**Figure 3:**
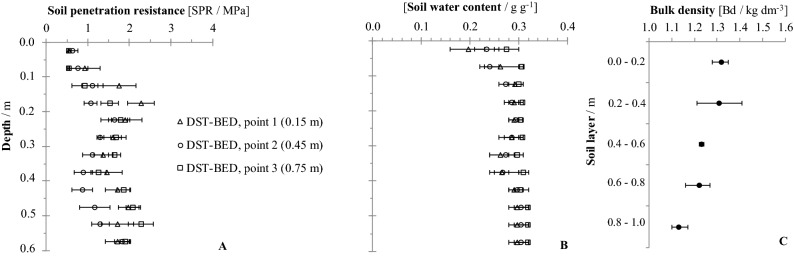
Soil penetration resistance (**A**) and soil water content (**B**) for deep strip tillage bed with no traffic (DST-BED) at three sampling positions (0.15, 0.45 and 0.75 m from planting row), and the average bulk density (**C**) at point 2. If confidence intervals do not overlap, the differences are significant (p ≤ 0.15).

**Figure 4:**
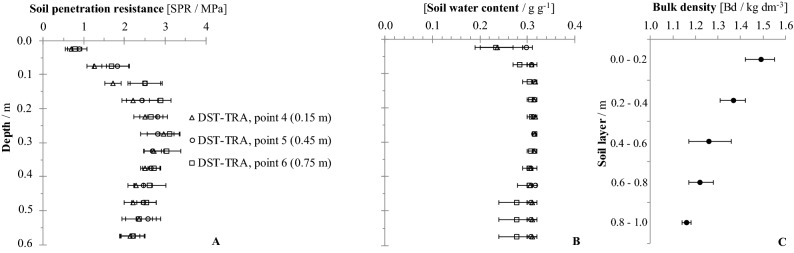
Soil penetration resistance (**A**) and soil water content (**B**) for the deep strip tillage traffic lane (DST-TRA) at the three sampling positions (0.15, 0.45, and 0.75 m from planting row)), and the average bulk density (**C**) at point 5. If confidence intervals do not overlap, the differences are significant (p ≤ 0.15).

**Figure 5:**
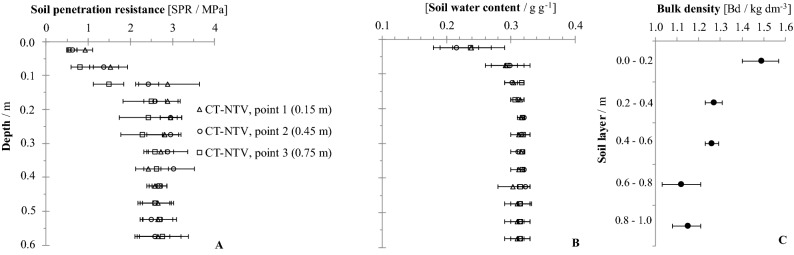
Soil penetration resistance (**A**) and soil water content (**B**) for the conventional tillage no traffic lane (CT-NTV) at the three sampling positions (0.15, 0.45, and 0.75 m from planting row), and the average bulk density (**C**) at point 2. If the confidence intervals do not overlap, the differences are significant (p ≤ 0.15).

**Figure 6:**
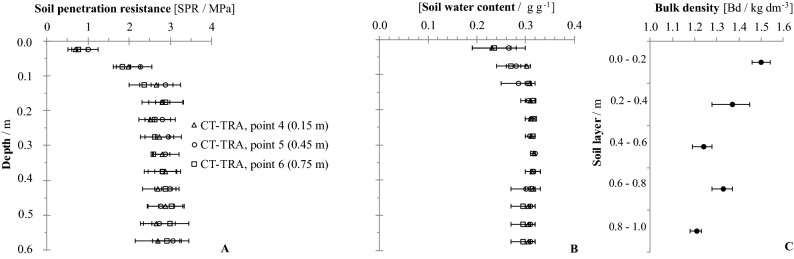
Soil penetration resistance (**A**) and soil water content (**B**) for the conventional tillage traffic lane (CT-TRA) at the three sampling positions (0.15, 0.45, and 0.75 m from planting row), and the average bulk density (**C**) at point 5. If the confidence intervals do not overlap, the differences are significant (p ≤ 0.15).

The mean values for the soil penetration resistance (SPR) under DST-BED (1.45 MPa) was lower than for the other treatments (2.55 MPa), indicating that the deep strip tillage had reduced the SPR (Fig. 2) to a depth of 0.6 m, having in mind that the subsoiling implement works up to 0.80 m. The soil water content (Figs. 3B, 4B, 5B, and 6B) revealed no significant differences among treatments. Thus, the lower SPR values at DST-BED are a result of soil structure alterations due to the soil tillage, and not affected by soil moisture. The SPR values for DST-TRA, CT-NTV and CT-TRA treatments presented no significant differences.

The SPR values above 2.0 MPa in the DST-TRA, CT-NTV, and CT-TRA at depths greater than 0.1 m indicate that there may be restrictions to root growth in these treatments, as observed by Otto et al.^30^ who studied sugarcane root growth on a Typic Kandiudox.

For the DST-BED treatment, there were significant differences in the comparing the sampling points in the 0.1–0.2 m layer (Fig. 3A), with the highest values at point 0.15 m from the sugarcane planting row. The higher SPR at this position indicates the ephemeral results achieved through the rotary hoeing of the soil. The soil particles could have reorganized since soil tillage^31^, especially at the sampling points closest to the sugarcane plants, through the combined effects of the formation of the furrows for the planting of the crop and the passage of the sugarcane cultivation machinery. Another cause of the acceleration of soil reorganization close to the plants includes the frequent cycles of soil drying and wetting around the roots^32^. Marasca et al.^8^ also verified higher SPR values near in-row deep tillage in relation on the center of the beds, agreeing with the results in the present study.

Regardless, for DST-BED, at depths of 0.4 m to 0.5 m (Fig. 3A), there are significant differences between the SPR values at the center of the bed (point ‘2’, 0.45 m) compared to the two positions within the bed adjacent to the ratoons (point ‘1’, 0.15 m and point ‘3’, 0.75 m). These differences could be attributed to residual effects of the vertical shanks of the subsoiler implement utilized in soil deep tillage, as observed by Marasca et al.^8^ who found SPR values below 1.0 MPa in the centers of the beds in the 0.30–0.45 m layer.

In the DST-TRA treatment (Fig. 4A), the lowest values for SPR were recorded at point ‘4’, 0.15 m from the ratoons, for the 0.1–0.2 m layer. These low values are the result of the soil tillage with rotary hoe equipment, which worked the soil over a width of 1.2 m, allied to the subsequent furrowing of the beds for the planting of the crop. Both operations produced soil disturbance, which extended into the lane set aside for agricultural traffic. At the two other sampling positions within the traffic lane, points 5 and 6, located 0.45 and 0.75 m, respectively, from the ratoons, the soil of the 0.1–0.2 m layer showed greater SPR values, with values of 2.5 MPa and 1.5 kg dm^-3^ for Bd (Fig. 4C).

Across the lane with no agricultural traffic and conventional tillage (CT-NTV), in the 0.05–0.15 m soil layer, there are significant small values for SPR registered for point ‘3’, 0.75 m from the ratoons (Fig. 5A). At that point, close to the next planting line, the furrowing operation broke and loosened the soil structure. Across the traffic lanes for conventional tillage (CT-TRA), there was no difference observed for the SPR values (Fig. 6A) among all layers.

### Bulk density

Bulk density (Bd) was evaluated at the center of the lanes, equivalent to points ‘2’ and ‘5’ (Fig. 1). There was no influence of soil tillage and machinery traffic on Bd below 0.60 m, with mean values close to 1.26 kg dm^-3^. For the DST-TRA, CT-NTV and CT-TRA treatments (Figs. 4C, 5C and 6C), the Bd was close to 1.50 kg dm^-3^ at 0.0–0.2 m. Only for the DST-BED treatment (Fig. 3C) was Bd lower (1.32 kg dm^-3^ at 0.0–0.2 m), because of the resulting soil surface structure disaggregation by rotary hoe tillage, which reached a 0.4 m working depth. Scarpare et al.^33^, studying the development of sugarcane under a conventional tillage system and deep strip tillage in a Haplustox (Oxisol) found bulk density values of 1.66 kg dm^-3^ in CT and 1.51 kg dm^-3^ in DST for the 0.0–0.2 m layer.

### Sugarcane root systems

**Figure 7:**
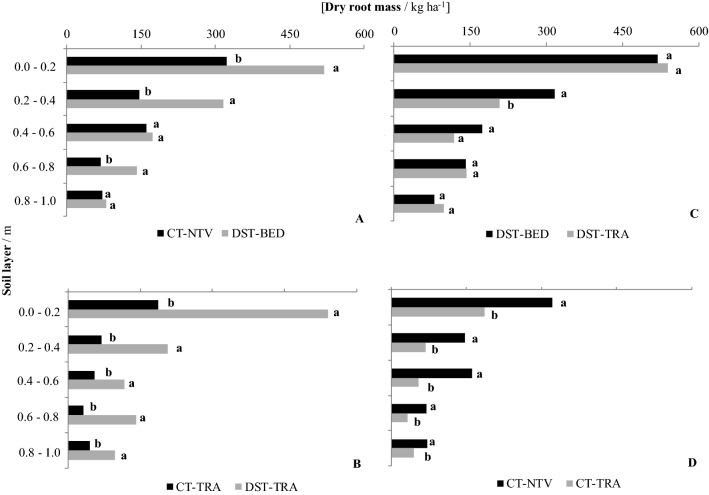
Dry root biomass per unit surface area for: (**A**) conventional tillage no traffic lane (CT-NTV) and deep strip tillage bed with no traffic (DST-BED), (**B**) conventional tillage traffic lane (CT-TRA) and deep strip tillage traffic lane (DST-TRA), (**C**) DST-BED and DTS-TRA, and (**D**) CT-NTV and CT-TRA. Different lower case letters indicate significant differences between treatments with the p ≤ 0.15 confidence intervals of the log-transformed data.

**Figure 8:**
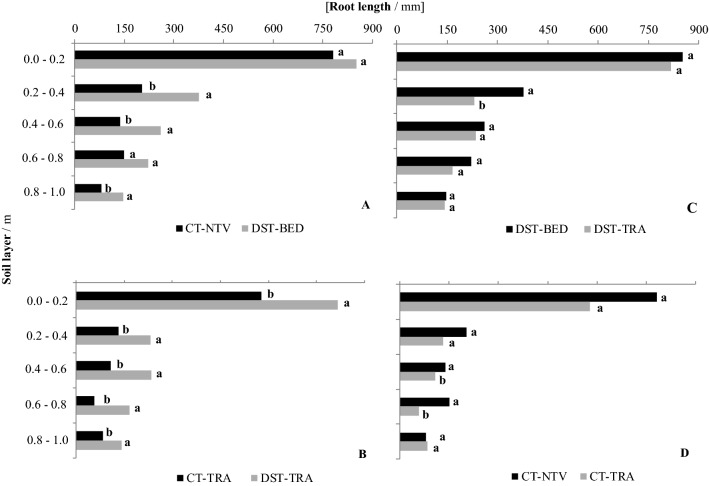
Root length for: (**A**) conventional tillage no traffic lane (CT-NTV) and deep strip tillage bed with no traffic (DST-BED), (**B**) conventional tillage traffic lane (CT-TRA) and deep strip tillage traffic lane (DST-TRA), (**C**) DST-BED and DTS-TRA, and (**D**) CT-NTV and CT-TRA. Different lower case letters indicate significant differences between treatments with the *p* ≤ 0.15 confidence intervals of the log-transformed data.

**Figure 9:**
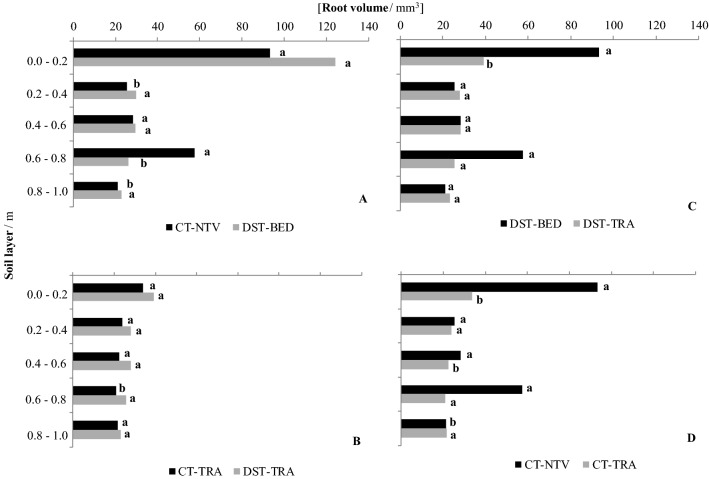
Root volume for: (**A**) conventional tillage no traffic lane (CT-NTV) and deep strip tillage bed with no traffic (DST-BED), (**B**) conventional tillage traffic lane (CT-TRA) and deep strip tillage traffic lane (DST-TRA), (**C**) DST-BED and DTS-TRA, and (**D**) CT-NTV and CT-TRA. Different lower case letters indicate significant differences between treatments with the *p* ≤ 0.15 confidence intervals of the log-transformed data.

The root systems of sugarcane explored a large volume of soil and showed an expected exponential decline with depth, as reported by Smith et al.^12^, for root biomass (Fig. 7), root length (Fig. 8), and root volume (Fig. 9). The concentration of roots and rhizomes (underground organs to retain plant nutrients) on the soil surface layers occurs because of the greater levels of soil fertility than at deeper depths^34^, which is consistent with results obtained by Cury et al.^35^ and Esteban et al.^36^ for an Oxisol.

### Dry root biomass

Comparing no traffic lanes for the 0.0–0.2, 0.2–0.4, and 0.6–0.8 m layers, the dry root biomass in the CT-NTV treatment was less than under DST-BED (Fig. 7A). The differences were substantial; there was 62% more dry root biomass in the 0.0–0.2 m layer of the DST-BED treatment compared to the CT-NTV, and 47% more dry root biomass for the 0.2–0.4 m layer.

Within the lanes for agricultural traffic (treatments DST-TRA and CT-TRA), for all layers, a greater dry root biomass was observed for the DST-TRA plot (Fig. 7B). For the 0.0–0.2 m layer, the CT-TRA dry root biomass was only 35% of the mass in DST-TRA.

A greater dry root mass for the DST-BED treatment in relation to DST-TRA was observed only for the 0.2–0.4 m soil layer (Fig. 7C), but there was a greater dry root mass for CT-NTV in relation to CT-TRA for all the evaluated layers (Fig. 7D).

This is a result of the better soil physical quality under DST-BED (Fig. 2) as also observed by Souza et al.^37^, and Souza et al.^38^ comparing sugarcane roots in areas with controlled traffic management.

Despite the similarity of the soil penetration resistance at depths under the traffic lanes (Fig. 2, DST-TRA, and CT-TRA), a greater dry root mass was found for the DST-TRA treatment in all of the layers (Fig. 7B). Considering the 0.0–0.2 m layer, the mean SPR for both CT-TRA and DST-TRA increases with depth, but for CT-TRA the increase is greater; it was 0.8 to 2.8 MPa compared to 0.8 to 2.4 MPa for DST-TRA, and the dry root mass for CT-TRA was 35% less than for DST-TRA.

### Root length

Higher dry root mass (Fig. 7) correlated with longer roots (Fig. 8). For the layers 0.2–0.4, 0.4–0.6, and 0.8–1.0 m, the root lengths for the DST-BED treatment were greater than for CT-NTV (Fig. 8A). In fact, Scarpare et al.^33^ also found higher values of sugarcane root length in DST. In addition, they showed a more homogeneous root distribution in the soil profile under DST, mainly below 0.6 m. Meanwhile in CT, the roots were concentrated near the planting line, and the results were attributed to a possible improvement of the soil physical properties provided by the soil with DST, such as lower compaction, lower bulk density, and higher soil porosity.

For all layers, the roots in the DST-TRA treatment were longer than those in the CT-TRA treatment (Fig. 8B). When comparing DST-BED with DST-TRA, the only layer with a significant difference in root lengths was 0.2–0.4 m (Fig. 8C). For the conventional tillage, longer roots in the lanes with no traffic (CT-NTV) compared to the lanes for agricultural traffic were only observed in the 0.4–0.6 and 0.6–0.8 m layers (Fig. 8D).

These results are related to the lower soil compaction indexes, already mentioned, which were also identified by Souza et al.^38^, who found 44% more dry biomass of roots down to 0.30 m and greater root areas down to 0.4 m, respectively, in the sugarcane beds in relation to the traffic lane. The results for root volume (Fig. 9) demonstrated that the root volumes were greater in DST-BED than in CT-NTV in the 0.2–0.4 and 0.8–1.0 m layers (Fig. 9A). Meanwhile, for the traffic lane, the only root volume difference was for the 0.6–0.8 m layer, where the volume was lower under the CT-TRA treatment (Fig. 9B). When comparing DST-BED with DST-TRA, the root volumes were only greater for the beds in the 0.0–0.2 m layer (Fig. 9C). There were differences between the root volumes in the CT-NTV and CT-TRA. For the 0.0–0.2 and 0.4–0.6 m layers, the greater volume was found in the CT-NTV, while the volume was greater for the CT-TRA in the 0.8–1.0 m layer (Fig. 9D).

### Root volume

Trends in the root volume values generally followed those for root lengths, with the largest volume values for DST-BED (Fig. 9A). Thus, the increase in surface area and root volume favors the development of plants shoots since it is possible to explore a higher soil volume^36^. The majority of the differences in root volumes were not statistically significant. Examination of the raw data showed that the root volumes, calculated from the length and diameter of each root, displayed great variability. Taking the root volumes for DST-BED as an example, the coefficient of variation of the root volumes was 205%, and the logarithmic transformation of the raw data {*y*(*v*) = log ([*v* / mm^3^] + 10)} reduced the coefficient of variation to 48%. However, this is still a large value, and according to Warrick & Nielsen^39^ corresponds to medium variability, sufficient to interfere with the sensitivity of statistical tests to compare means.

Although it had high variability, the root volume for the CT-NTV was larger than for DST-BED in the 0.6–0.8 m soil layer (Fig. 9A). For this same layer (0.6–0.8 m) the CT-NTV and DST-BED were equal in root length at 0.6–0.8 (Fig. 8A). This larger root volume for the CT-NTV for the same root length point to great roots diameters. In the sugarcane root system, root diameters can differ markedly^40^. As soil penetration resistance is great in CT-NTV compared to DST-BED, especially at depths below 0.1 m (Fig. 2), root growth could be reduced and present morphological and anatomical modifications becoming thicker, and so of greater volume^41^.

The increase in soil compaction has been associated with changes in the structural and morphological aspects of roots. Queiroz-Voltan et al.^42^ found that increased bulk density in a clayey Rhodic Kandiudox in Brazil increased the cortical thickness/vascular cylinder thickness ratio in sugarcane roots. Alameda et al.^43^ have reported that responses of plants to soil compaction are primarily manifested by changes in the root characteristics, as decrease in length and number of fine roots.

## Discussion

The soil on traffic control lanes along which there was no agricultural traffic (CT-NTV and DTS-BED) was not subject to pressures from machines. However, although the SPR values were higher along the traffic lane, CT-NTV did not differ significantly from CT-TRA, and these treatments did not differ from DTS-TRA.

DST-TRA represents the experimental initial condition of the soil compaction, as it was not tilled, and this treatment was expected to have the highest values for Bd and SPR compared to CT-TRA and CT-NVT treatments, for which the whole area was prepared with disk harrows. Below 0.1 m, SPR values of up to 2.5 MPa in DST-TRA, CT-TRA and CT_NVT were the result of soil compaction in previous sugarcane cycles. This finding is in agreement with Costa et al.^44^, who found SPR values of 2.52 MPa in the 0.2–0.4 m layer in a Rhodic Kandiudox under CT cultivated with sugarcane. Chang et al.^45^ also called attention the underlying compaction generated by previous agricultural practices. The SPR and Bd values for CT-NTV and CT-TRA indicate that the CT system was not able to reverse the increment of SPR that occurred at previous over the period that the experimental area has been under cultivation with sugarcane. The high SPR values in subsurface layers are difficult, costly, and time consuming to remedy.

On CV, topsoil mobilization with disk harrows disrupted the surface structure of the soil but increased the SPR levels on subsurface through the cumulative effects of the pressures on the soil resulting from the passage of the associated machinery. Mechanical actions of agricultural implements on operations such as soil tillage and management, and the traffic for sowing, cultivation, and harvesting of the sugarcane resulted in soil degradation. Torres et al.^46^ also detected degradation of soil physical properties under conventional tillage for sugarcane production, with a compacted layer between 0.10 and 0.30 m in depth after the fifth and sixth ratoon crop. According to a related study by Silva & Cabeda^47^, the use of harrows in soil tillage operations contributes to the compaction of soil well below the surface. The superficial destruction of the soil structure renders its aggregates susceptible to rupture, facilitating the transmission of the pressures exerted by the disc harrow to deeper layers of the soil, because the less compacted the soil is, the greater its susceptibility to compaction.

Intense disaggregation on soil surface has made the soil more prone to the transmission of loads arising from mechanized operations into the deeper layers of the soil^1^, thereby favoring compaction in these layers^48^. The effect of traffic on conventional tillage is mainly accumulated on the surface^49^.

The resulting compaction of the soil under the layer disturbed during soil tillage in CT-TRA resulted in the formation of a distinct compacted layer at 0.2 m. Similar observations of the formation of a compacted layer have been reported by Otto et al.^30^.

Souza et al.^37^ observed the greatest soil compaction under the permanent lines followed by the agricultural traffic through a sugarcane crop. In the present study, differences between the soil physical attributes of CT-NTV and CT-TRA treatments were not discernible. The single cycle was probably not sufficient to produce significant additional compaction under CT-TRA compared to CT-NTV.

In general, soil tillage by deep strip tillage provided better physical conditions for the development of the root systems of the sugarcane crop than conventional tillage, where the roots encountered soil layers that hindered their growth. For the DST-BED treatment, the deep soil tillage resulted in lower SPR values, producing better physical conditions for penetration by the roots. Scarpare et al.^33^, also in a study with sugarcane, found a higher concentration of roots in the bed of the DST in relation to the no traffic lane of the CT. The soil was considered to have undergone structural alleviation, leading to an increase in porosity and conditions propitious to the horizontal and vertical growth of the roots.

A previous study of the effects of soil compaction on sugarcane root systems by Souza et al.^37^ found lower densities of roots where machine traffic had been more intense and associated these observations to physical limitations on root growth caused by the transit of machines over the soil. Faroni & Trivelin^49^ observed a reduction of 91% in root concentration for the 0.0–0.2 m layer at distances from the planting lines corresponding to agricultural traffic lanes. Thus, under both soil tillage systems, less root growth was expected in the traffic lanes. However, neither the total masses nor the lengths of the roots were significantly different for DST-TRA compared to DST-BED, so any effect of traffic on these root attributes for the plot prepared by DST was weak, indicating that the mechanisms involved in the physical properties and root growth of sugarcane under deep strip tillage are complex and sensitive to many factors^8,31^.

The present study has examined the first cropping cycle of a planting of sugarcane in a Rhodic Kandiudox. It is evident that the deep strip tillage system of soil management has provided better physical conditions for the development of the roots than conventional tillage. However, since the study was limited to the first cycle and a single class of soil, the information generated is not considered sufficient for decision-making. There is a need for further research directed at the detection of the long-term effects resulting from the adoption of deep strip tillage for the cultivation of sugarcane as well as to examine the application of deep strip tillage to other soil classes.

## Conclusions

1. Traffic lanes were characterized by a greater resistance to penetration and shorter root system compared to lanes without traffic, independent of the system of soil tillage.
2. The Deep Strip Tillage induced a significant difference in resistance to penetration however root growth was not restricted to beds and no traffic lines.
3. The Conventional Tillage was not able to reverse the high soil resistance that resulted from the previous period when the experimental area had been under sugarcane cultivation.
